# How does childhood socioeconomic hardship affect reproductive strategy? Pathways of development

**DOI:** 10.1002/ajhb.22793

**Published:** 2015-09-26

**Authors:** Paula Sheppard, Mark S. Pearce, Rebecca Sear

**Affiliations:** ^1^ Department of Population HealthLondon School of Hygiene & Tropical MedicineLondonWC1E 7HTUnited Kingdom; ^2^ Institute of Health & Society, Newcastle University, Newcastle upon Tyne, Tyne and Wear NE1 7RUUnited Kingdom

## Abstract

**Objectives:**

In high‐income populations, evidence suggests that socioeconomic disadvantage early in life is correlated with reproductive strategy. Children growing up in unfavorable rearing environments tend to experience earlier sexual maturity and first births. Earlier first births may be associated with higher fertility, but links between socioeconomic disadvantage and larger family size have rarely been tested. The pathways through which early disadvantage influences reproduction are unknown. We test whether physiological factors link childhood adversity to age at first birth and total children.

**Methods:**

Using data from the Newcastle Thousand Families Study, a 1947 British birth cohort, we developed path models to identify possible physiological traits linking childhood socioeconomic status, and poor housing standards, to two reproductive outcomes: age at first birth and total children. We explored birth weight, weight gain after birth, childhood illnesses, body mass index at age 9, age at menarche, and adult height as possible mediators.

**Results:**

We found direct, negative effects of socioeconomic status (SES) and housing on age at first birth, and of housing on fertility. Although we found links between childhood disadvantage and menarche and height, neither of these were significantly correlated with either reproductive outcome. Age at first birth completely mediates the relationship between childhood adversity and total fertility, which we believe has not been empirically demonstrated before.

**Conclusions:**

While there are some links between childhood adversity and child health, we find little evidence that physiological pathways, such as child health and growth, link early childhood adversity to reproductive outcomes in this relatively well‐nourished population. Am. J. Hum. Biol. 28:356–363, 2016. © 2015 The Authors American Journal of Human Biology Published by Wiley Periodicals, Inc.

It is well documented that in high‐income populations, childhood adversity influences reproductive strategy in later life by accelerating events such as timing of puberty and first birth (Alvergne et al., 2008; Belsky et al., 1991; Blell et al., 2008; Chisholm et al., 2005; Ellis, 2004; Nettle, 2010; Nettle et al., 2011). Life history theory (LHT), which concerns the allocation of energy across the life course, and the resultant trade‐offs that occur between growth, reproduction, and somatic maintenance suggests that this is an adaptive strategy. Hardship in childhood due to suboptimal rearing conditions may be an indicator of higher local mortality risks. According to LHT, this would favor faster pubertal maturity, and earlier first birth, so as to offset the risk of death before successful reproduction, which requires raising children to an age where they are able to reproduce themselves, can be completed (Chisholm, 1993; Ellis, 2004; Nettle, 2010; Promislow and Harvey, 1990). Such a “fast” life history strategy may also be associated with opting for quantity over quality of offspring, resulting in higher numbers of children who are each conferred with lowered parental investment (Belsky et al., 1991; Chisholm, 1993; Stearns, 1992; Trivers, 2002). Shortened expected lifespan is a plausible ultimate explanation for why it is adaptive to reproduce younger, and produce more offspring (given the increased risk of death of any offspring produced), than those from low mortality environments. Furthermore, if adverse conditions during childhood have a lasting effect on an individual's health, then even if external (local) mortality rates are relatively low, one's own healthy life expectancy may be directly jeopardized, making it adaptive to adopt a faster reproductive strategy (Nettle et al., 2013b; Rickard et al., 2014).

While theory suggests that fast life histories may also be associated with a greater number of children produced, this has rarely been tested, at least in high‐income contexts (although see Nettle, 2010 for an indirect test supporting this hypothesis). Further, little has been done to try and ascertain what it is about childhood hardship that seems to accelerate reproductive timing in such settings. One exception is Nettle et al. (2011) who tested whether the relationship between early life adversity (socioeconomic position, low parental involvement, unstable home address, and duration of breastfeeding) and age at first pregnancy among British women was mediated by behavioral and emotional problems at age 11. They found only weak evidence that the relationship was mediated by variables that are arguably indicators of psychosocial stress, yet a robust direct effect remained between the childhood adversity and age at first pregnancy. Therefore, it is reasonable that physiological traits may be more convincing mediators linking early life economic adversity and reproductive outcomes later in life.

In the current study, we explore potential physiological pathways that may link early life adversity with two reproductive outcomes later in life: age at first birth and total number of surviving children, using data from a cohort of women born in 1947 in Newcastle upon Tyne, UK. While many studies look at age at first birth or pregnancy as a common life history outcome, few have the ability to explore the longer term effect on total fertility. These data have been collected over 50 years and thus allow us to do both. We further examine the relationship between age at first birth and total fertility, as there is evidence to suggest that this relationship is fairly robust in both high (Borgerhoff Mulder, 1989) and low fertility (Helle, 2008; Trussell and Menken, 1978) contexts, even though high levels of contraceptive use can potentially break the link between the timing of reproduction and how many children are produced overall. We predict that any early life factors that are associated with one of these outcomes are, therefore, likely to have a similar association with the other. This longitudinal survey collected very rich data on childhood physiological and health outcomes, allowing a detailed study of a number of possible variables which might mediate these relationships. We describe these in detail below.

## MEASURES OF CHILDHOOD DISADVANTAGE

The current study uses two measures of childhood socioeconomic disadvantage, measured at the individual level: low paternal occupational status (a common measure of SES at this time) and sub‐standard housing conditions at birth. We analyze these two separate factors in an attempt to tease apart whether, and how, specific elements of childhood adversity may influence later outcomes and through which pathways.

### Socioeconomic status

In high‐income settings, lower SES is correlated with shortened life expectancy, mediated by poverty, poor education, unemployment, behaviors associated with increased health risks (e.g., smoking), and actual poor health (even in childhood) (Blumenshine et al., 2010; Lin et al., 2003; Stronks et al., 1997). Therefore, we predict that low SES will be linked to a faster reproductive strategy, operationalized as earlier age at menarche, earlier first birth, and more children. A previous study among contemporary British women tested this hypothesis and found that neighborhood SES (as indicated by a multiple deprivation index) was associated with earlier reproduction, and higher rates of reproduction (measured indirectly) (Nettle, 2010). Links between neighborhood SES at birth and age at first motherhood in that population appeared to be mediated by low birth weight and low paternal involvement (Nettle et al., 2010). Such results support the hypothesis that low SES will be linked to faster life history strategy, although neighborhood characteristics may be proxies for local mortality rates, they are not necessarily good indicators of individual SES during childhood. The timing of reproduction and reproduction rates were also measured as neighborhood averages. In the current study, we avoid limitations associated with using area‐level proxies by using individual level SES measures and life history outcomes.

### Housing conditions

We consider poor housing standards as a second measure of childhood adversity; an indicator of the physical childhood environment. While this is likely to be somewhat correlated with SES, SES may not necessarily be perfectly correlated with the condition of the housing. Poor housing conditions during childhood are known to be associated with poor health, even if the individual moves to improved housing later in life (Marsh et al., 1999). Inadequate housing is often considered to be partly a product of overcrowding (as it is in the current study, Pearce et al., 2009) which is in itself also associated with ill health (Bansal and Saxena, 2002). Other aspects of housing such as structural deficiency and lack of access to plumbed amenities indicates an impoverished childhood environment which, as outlined in the previous section, is expected to be associated with most covariates in our models. Poor housing conditions, in particular overcrowding and lack of adequate plumbing, are likely to be associated with ill child health, which may in turn affect growth. As far as we are aware, the relationship between poor housing conditions, a measure of the physical childhood environment, and reproductive strategies has not been explored before.

## POTENTIAL PATHWAYS

There are a number of plausible physiological traits that link early life adversity and faster reproductive strategy. Growth is likely to be affected by childhood health outcomes which are in turn expected to be a direct product of being reared in a harsh environment. Therefore, indicators of growth and development, such as age at menarche and height, are likely to be correlated with the timing of first reproduction, and total reproductive output, because markers of poor childhood health may indicate shorter life expectancy.

### Markers of childhood health

In the current study, we look at an array of physiological and health factors during early childhood, which may affect development and reproductive strategies. We propose that birth weight, rate of growth during infancy, and infant health would affect body mass index (BMI) and age at menarche. Similarly, childhood health should have an impact on adult height. Heavier birth weight has previously been shown to predict taller adult height (Sorensen et al., 1999), later age at menarche (Adair, 2001; Romundstad et al., 2003), and higher BMI during adolescence (Romundstad et al., 2003). It is likely that chronic illness during childhood is a product of poor housing conditions, poverty, and increased exposure to pathogens in overcrowded environments. Prolonged illness during childhood has also been linked to later onset of menarche (although this was in nutritionally stressed rural Guatemala) (Khan et al., 1996) and shorter adult stature (Beard and Blaser, 2002; Crimmins and Finch, 2006).

Numerous previous studies have identified relationships between childhood SES and measures of childhood height or weight. A study among Dutch schoolchildren found that children from low SES neighborhoods were on average shorter than those from more affluent ones (Jansen and Hazebroek‐Kampschreur, 1997); population‐level associations demonstrating the same relationship between low SES and short height have been found in a study of eight low and middle income countries (Beard and Blaser, 2002). Low SES in high‐income populations, where over‐nutrition is more of problem than under‐nutrition, also tends to be associated with higher levels of obesity (Cecil et al. 2005; McLaren 2007; Sobal and Stunkard, 1989). These findings suggest that the variables we use are good candidates for physiological health‐related traits linking early life disadvantage and reproductive strategy.

In general, we expect that children who experience harsh early childhoods will follow a faster reproductive strategy than those from more benign childhood environments. Therefore, we predict that high levels of childhood disadvantage will lead to lower birth weight, faster infant weight gain, and poorer health during the first year of life. These factors should in turn be associated with poor growth resulting in shorter stature, and earlier menarche, which is also associated with higher BMI during middle childhood (Nettle et al., 2013a). The reason that lower birth weight should be associated with higher BMI (but not taller stature) is because undernourished newborns tend to adopt a catch‐up growth strategy, which is associated with insulin resistance, more central adiposity (visceral fat), and other factors related to obesity (Ong et al., 2000). It is also possible that underweight neonates lack muscle mass and will gain a higher proportion of fatty mass during a post‐partum growth spurt (Eriksson et al., 2001). On the other hand, heavier newborns who end up with high BMIs may have a higher lean mass to fat mass ratio which is less problematic in terms of health outcomes in later life (e.g., coronary heart disease and type 2 diabetes) (Kahn et al., 2000). The relationship between birth weight, BMI, and height is clearly not simple and requires careful interpretation.

### Puberty (age at menarche)

Age at puberty is a good candidate as a mediator between childhood adversity and reproduction, even in contracepting populations. First, this is because reproduction cannot start without reaching puberty; second, because women who start puberty younger are likely to be embarking on a fast life history course that includes early reproduction and possibly higher numbers of children (Ellis, 2004). In high‐income populations where most women do not start reproduction soon after first menstruation (like many natural fertility populations), there is likely to be a longer time period between these two events. However, there is still a relationship between earlier puberty and early first birth, albeit not as close, in both high‐income settings (Udry, 1979; Udry and Cliquet, 1982) as well as in more traditional, high‐fertility settings (Borgerhoff Mulder, 1989; Udry and Cliquet, 1982).

In the sample we use, this pattern is demonstrated (Table 1). Disadvantaged SES women have earlier ages at puberty and first births and they also have a shorter interval between age at puberty and age at first birth than more advantaged SES women (although there is little difference between medium SES women and the most advantaged SES women).

**Table 1:** Distribution of mean ages at menarche and first birth (years) stratified by SES

SES	Mean age at menarche	Difference	Mean age at first birth
Lowest tier	12.58	9.06	21.64
Medium	13.07	11.90	24.97
Highest tier	13.23	11.34	24.57

The relationship between SES and age at puberty is context dependent and in some contexts, likely to influenced by access to nutrition. In low‐ and middle‐income settings, this relationship is usually reported as negative with higher SES women maturing earlier (Adair, 2001; Sorensen et al., 1999). However in high‐income settings, the pattern is less clear; low SES is sometimes correlated with earlier puberty (James‐Todd et al., 2010; Ellis and Essex, 2007), although in some cases there is no evidence for an association either way (Blell et al., 2008; Ersoy et al., 2004; Moffitt et al., 1992). Post‐war Newcastle upon Tyne was arguably a middle‐income setting and continued post‐war food rationing (until 1954) would also have affected levels of nutrition received by all social classes.

Less is known about the relationship between age at menarche and total numbers of children in high‐income contexts, but there is a positive association between age at menarche and total surviving offspring among the Kenyan Kipsigis (Borgerhoff Mulder, 1989). Since age at first birth is related to completed fertility (Kohler et al., 2001), we would expect earlier menarche to be associated with both younger age at first birth and higher numbers of children.

### Height

We chose height as a mediating variable between early life adversity and reproduction because taller height is an indicator of a longer period of growth, and therefore, an extended childhood. Research has shown that taller adult height is associated with later first births in 1960s Finland (Helle, 2008), contemporary United States (Stulp et al., 2012), and in the Gambia (Sear et al., 2004). These findings provide empirical evidence for the predicted trade‐off between growth and reproduction, that is, that women who spend a longer time growing postpone first births compared with women who stop growing younger and divert somatic resources toward reproduction (Sear, 2010). With regard to the relationship between height and total fertility, the picture is less clear and appears to be heavily context dependent (Sear, 2010). Cross‐cultural reviews have reported that sometimes, taller women have higher numbers of children while in other cases shorter women do, particularly in low income settings. In high‐income contexts, there appears to be little correlation between these two variables (Sear, 2010). Often these relationships are attributable to local ecological factors (Sear, 2010; Stulp et al., 2012). If taller women do have later first births, we might predict then that, on average, they would produce fewer children.

## METHODS

### Data

The Newcastle Thousand Families Study (NTFS) came about in response to the relatively high infant mortality rate in Newcastle upon Tyne prior to the Second World War, compared to most other parts of the United Kingdom. For every thousand live births, 62 infant deaths were recorded in 1939. The study was postponed because of the war, but in 1947, the study began. All but four of the 1146 children born in Newcastle upon Tyne in May and June of that year were recruited and followed intensely for the first five years of the child's life (*n* = 1142). This initially included weekly visits by health visitors and the study field workers, enabling an in‐depth assessment of the child's health and social circumstances including information on the parents and the state of the housing the child inhabited. During later years, the children were intermittently followed up until age 15, creating a longitudinal study. In 1996‐1998, when the respondents were aged 49 to 51 years, another follow‐up traced 89% of the original surviving sample (99 individuals had died): 574 (55%), 316 of whom were women, returned questionnaires supplying retrospective health and demographic information. These participants are representative of the original cohort sample, for a detailed cohort profile see Pearce et al (2009).

In the present study, we eliminated twins (*n* = 5) as children who share a womb are small at birth, may have subsequent health problems, and are not statistically independent data points. We dropped men from the sample, as we only have puberty data for women. We also eliminated all those women who did not provide information on age at first birth, total number of live births, and number of surviving children in 1997 (about 25% of those who were traced), which left us with a sample size of 251 women. Although the sample is not big, the NTFS provides comprehensive health histories of the participants which allows for more detailed analysis than usual and has information on completed fertility due to the long length of follow‐up.

### Variables

#### Childhood adversity measures

*Socioeconomic status* of the child's father was measured using the Registrar General's occupational classificatory system comprising five social classes, with one assumed to represent the most advantaged (Spence et al., 1954) (summarized in Table 2). *Housing grade* was determined by the Deputy Chief Sanitary Inspector who visited and assessed the housing standards of four‐fifths of the respondents (Spence et al., 1954). Houses were graded according to the level of overcrowding, structural adequacy, shared/lack of amenities (lavatory, bath, no bath access), and availability of hot water. A score was derived by adding up the number of problems each house had, ranging from zero to all four, so a high score indicates poor housing.

**Table 2:** Means and standard deviations for all variables

Variable	Mean (SD)
Socioeconomic status (1 = high)	3.3 (0.88)
Housing grade (0 = no problems)	1.01 (1.12)
Birth weight (kg)	3.38 (0.51)
Percent change in weight from birth to 6 months	120 (47)
Ailments reported during first year	1.42 (1.45)
Body mass index at age 9	16.30 (2)
Age at menarche (years)	12.95 (1.55)
Adult height (cm)	161.46 (6.07)
Age at first birth (years)	23.95 (4.9)
Total number of surviving children	2.22 (0.85)

#### Mediating variables

*Birth weight* was measured at birth, in kilograms. *Weight gain from birth to 6 months* was calculated by subtracting birth weight from the infant's weight as measured at six months and dividing the answer by birth weight. This gives a measure of weight gain relative to birth weight, that is, the percent change in weight. The health variable is the number of recorded *ailments* the child suffered during the first year after birth and was calculated by adding up the number illnesses and infections reported on the child's medical records, as held by the study. Height and weight were measured at age nine by the school health service and *body mass index* was calculated from those figures. *Adult height* was self‐reported at age 50 measured in centimeters. *Age at menarche* was collected retrospectively, in 1997, and is recorded in years and months. We appreciate that these data were collected a long time after these women would have had their first menstruation but previous research has shown that recall of this event tends to be accurate to within a year, even 40 years after the event (Bean et al., 1979).

#### Dependent variables

*Age at first birth* and *surviving number of children* were also recorded retrospectively in the 1997 follow‐up questionnaire (Pearce et al., 2009). We model surviving children rather than live births, as we are interested in fitness, but the two variables differ only slightly, as child mortality was very low in this sample (8 deaths to 251 women). We only have birth history data for women who did reproduce, and so we are unable to take into account childless women, or the reasons for being childless. However, only 12% of British women born in 1947 remained childless by age 45 (Office for National Statistics, 2011).

### Analysis

A pair‐wise correlation matrix shows bivariate associations between all the variables in the study (Table 3). Path analyses were then used to examine the potential pathways between early life adversity and age at first birth and number of surviving children. In a final model, we include both age at first birth and total surviving children to test the strength of the link between these two variables. Path analysis is a series of multivariate regression analyses, performed simultaneously, to provide estimates and significance tests of hypothesized causal pathways between variables.

**Table 3:** Pair‐wise correlation matrix showing bivariate associations

	Housing grade scale 1947	Social class of father 1947	Birth weight in kilograms	% Weight gain 0–6 months	Number of ailments	BMI at age 9	Age at menarche	Adult height	Age at first birth	Surviving children 1997
Housing grade scale 1947	1									
Social class of father 1947	0.29*	1								
Birth weight in kg	−0.06	−0.07	1							
% Weight gain 0–6 months	−0.07	−0.04	−0.56*	1						
Number of ailments (0–1 year)	0.08	0.07	0.05	−0.09	1					
BMI at age 9	0.02	−0.17*	0.01	0.06	0.01	1				
Age at menarche	−0.02	−0.17*	0.12	−0.08	0.01	−0.25*	1			
Adult height	−0.09	0.02	0.27*	−0.02	−0.20*	−0.11	0.24*	1		
Age at first birth	−0.20*	−0.26*	0.06	0.02	−0.04	−0.02	0.13	0.07	1	
Surviving children 1997	0.15*	0.06	0.03	−0.06	−0.02	−0.10	0.03	0.09	−0.36*	1

*P < 0.05.

Path analysis is constrained by only allowing normally distributed error terms of the endogenous and outcome variables. The variable ailments is somewhat problematic as, although it is relatively normally distributed from 1 to 8, it is zero inflated. Modelling non‐normally distributed variables in path analysis biases the standard errors and so to minimize this bias, we used the quasi maximum likelihood method which provides robust standard errors. Full‐Information Maximum Likelihood (FIML) was used to deal with missing data from endogenous variables and to keep our sample size as high as possible. Similarly, number of children is also a count variable that should be modelled using Poisson regression, however, we are treating it as continuous because using FIML does not allow for modelling different error distributions within SEM and we would rather retain the maximum sample size allowed by the data. Our model fit statistic is presented as a coefficient of determination (CD), interpreted much like an R^2^ (Hooper et al. 2008; StataCorp 2011) because the use of robust standard errors prevents calculation of typically used fit statistics such as root mean square error of approximation (RMSEA).

We conducted three path analyses: (1) showing the proposed pathways between early life adversity and age at first birth, (2) the same diagram, but with paths leading to total numbers of children, and (3) including both reproductive outcomes in sequence with age at first birth predicting total numbers of children.

## RESULTS

Both measures of childhood socioeconomic disadvantage are positively correlated with one another, although the correlation coefficients are fairly low. This suggests that, while they are each measures of childhood adversity, they capture different aspects of such hardship. Our two reproductive outcomes are negatively correlated (earlier births = more children), which we would expect if they are both measures of a faster life history strategy. In this bivariate matrix, there are few correlations between our measures of childhood adversity and child health and growth; although lower social class is correlated with lower BMI at age 9 and with earlier menarche. Correlation coefficients also tend to be very small, suggesting the lack of statistical significance is not due to small sample sizes.

**Figure 1:**
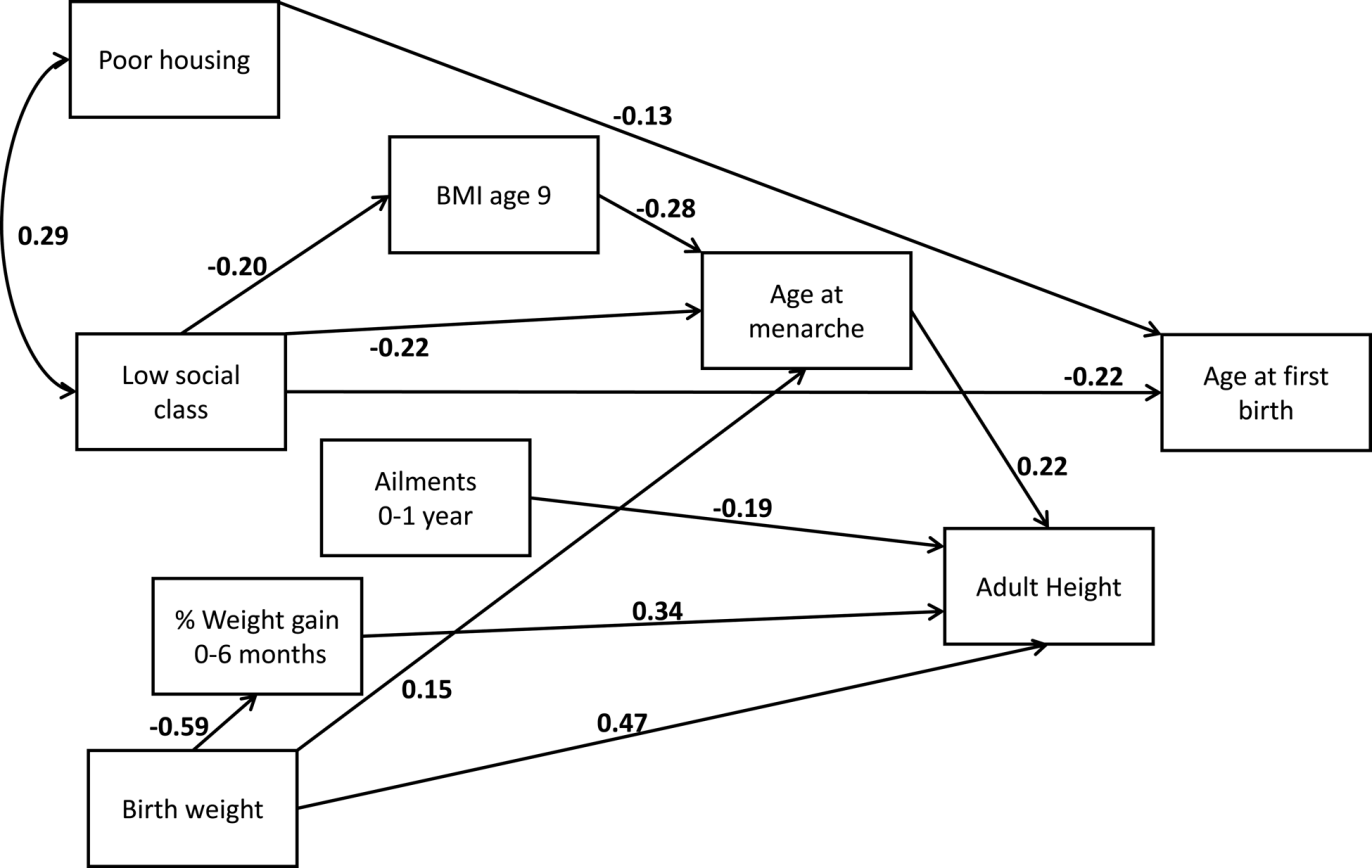
Path diagram showing statistically significant (*P* < 0.05) pathways between adversity at birth and age at first birth. Standardized beta coefficients are given. The model was fitted with robust standard errors; error terms are omitted for simplicity. Fit statistic: CD = 0.194.

**Figure 2:**
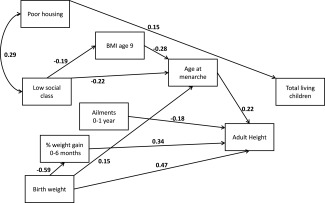
Path diagram showing statistically significant (*P* < 0.05) pathways between adversity at birth and total surviving children. Standardized beta coefficients are given. The model was fitted with robust standard errors; error terms are omitted for simplicity. Fit statistic: CD = 0.143.

**Figure 3:**
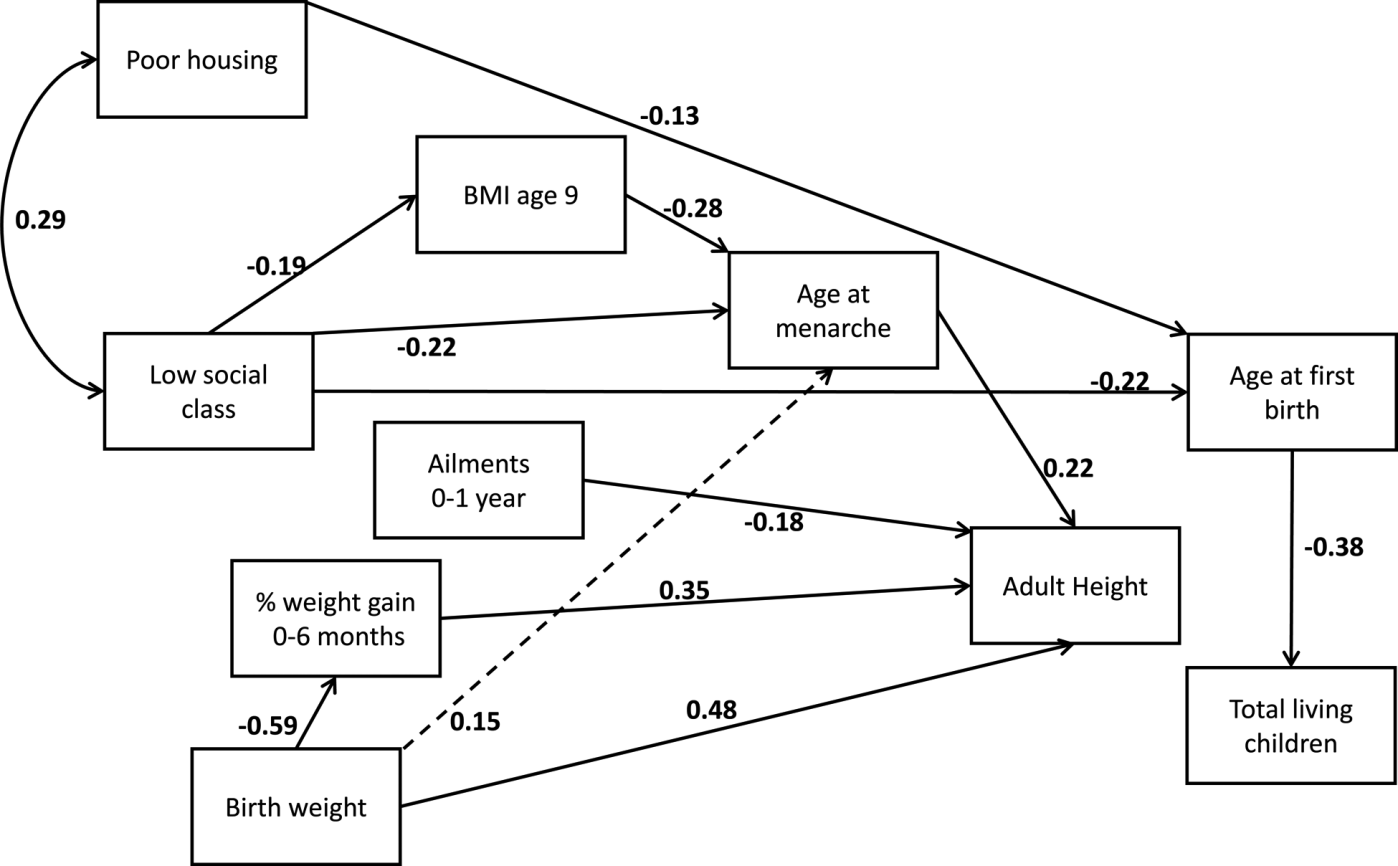
Path diagram showing statistically significant (*P* < 0.05) pathways between adversity at birth and age at first birth AND total surviving children. The dotted line between birth weight and age at menarche is now significant only at P = 0.051. The model was fitted with robust standard errors; error terms are omitted for simplicity. Fit statistic: CD = 0.185.

Figures 1, 2, 3 are path diagrams showing only the statistically significant pathways. These were modelled with pathways between *all* chronologically logical variables, but only those that are significant are shown, to simplify presentation of the models. Full models with both significant and nonsignificant estimates are provided in the Supporting Information Tables S1a, S1b, and S1c.

### Age at first birth

Our model supports previous studies demonstrating that childhood adversity is correlated with earlier first births. Low social class and poor housing conditions are both directly linked to age at first birth. No pathways through the mediating variables are indicated in our model, however. Low occupational status is linked to earlier age at menarche, both directly and through BMI, but there is then no correlation between age at menarche and age at first birth. Birth weight, directly and via weight gain, illnesses during infancy, and age at menarche are all correlated with adult height in the predicted directions (higher birth weight, greater weight gain, fewer ailments, and later menarche are all correlated with taller adult height), but height also does not appear to be associated with age at first birth.

### Total number of surviving children

Here we found a direct positive effect of poor housing on total fertility (surviving children), but not of social class, and again we find no pathways through which childhood adversity may link to this reproductive outcome (Fig. 2).

### Age at first birth and total surviving children

In the third model, we included both age at first birth and total surviving children and demonstrate a relationship between these two variables (Fig. 3). The effect of housing on total children becomes nonsignificant once age at first birth is included in the model, indicating that poor housing is correlated with higher numbers of children, but only indirectly, by way of early first birth. Otherwise, our model is very similar to our previous model: none of the potentially mediating factors are linked to either fertility outcome.

## DISCUSSION

Overall, we have confirmed previous results showing a correlation between greater socioeconomic disadvantage in childhood and earlier first births. We further find evidence that women who suffered greater childhood adversity also had higher numbers of children, but that this relationship is fully mediated by age at first birth. As far as we are aware, this has not been tested before. However, we do not find convincing evidence that these effects are mediated through childhood physiological factors (i.e., health or growth). Disadvantaged SES results in lower BMI at age nine, and earlier age at menarche, although higher BMI is associated with earlier menarche. In other words, the effect of social class on age at menarche is only slightly tempered by BMI, suggesting that SES is not indicative of nutritional status in this sample and may be a better indicator of psychosocial stress. Post‐war food rationing may partly explain this. The British public experienced controlled access to meat, dairy, and sugar (among other things) right up until 1954 and as such nutritional status became less SES‐dependent. The direct effect of SES on reproductive outcomes, therefore, is likely to indicate a stronger role of the social dimension of SES.

Later menarche is nevertheless associated with taller stature, possibly indicating a prolonged childhood, if adult height is a product of a longer childhood growth period. It is puzzling, therefore, that we find no correlations between age at menarche or adult height, and age at first birth or total number of children. It is possible that the relationship between longer childhood and later reproduction is not physiological and may instead be driven by psychological or behavioral characteristics or indeed, that there are interactions between two or more of those factors. Investment in embodied capital, which is not physiological (e.g., spending longer in education), could be one such trait (unmeasured in this study, although only around 10% of British women born in 1947 completed tertiary‐level education (Ratcliffe & Smith 2008)). Other research has shown that in this population, women who attained high IQ test scores during childhood also did not go on to enroll at university (Forrest et al. 2011). However, investment in physiological and nonphysiological embodied capital is likely to be positively correlated (those with greater resources available invest both in physical growth and nonphysical or cognitive development). Another point to note is that we were not able to include childless women in the study, which may have been more revealing. On the basis of national statistics, however, we would only expect two or three women to have been childless in this sample, and so we are confident that our results would not have been substantively different.

The psychological literature suggests that psychosocial stress may be an important mechanism linking early adversity with later reproductive outcomes (Belsky et al. 2007; Belsky et al. 1991). Our lack of evidence for physiological pathways here may provide very indirect evidence supporting this hypothesis, though unfortunately we are not able to test whether psychosocial stress is important here. Nevertheless, we might also expect psychosocial stress to influence physiological health and development (Wright et al. 1998; Lucini et al. 2005). One study that did attempt to test this found that the direct effects of adversity were much stronger than the psychosocial mediators they used (emotional and behavioral problems) on age at first pregnancy among contemporary British women (Nettle et al. 2011). Another study using UK data compared both behavioral and physiological traits of teenage mothers matched with a control group of women on SES background, age, and gestational age (Nettle et al., 2013a). Their findings show differences in both types of traits—physiological and behavioral—between the two groups of women, however, as the analysis only showed differences between the two groups for each variable singly, we have no way of knowing the relative strength of each trait or if some of the apparent differences fall away once controlling for others. Further research that examines all of these types of outcomes and interactions between them, in the same analysis, is needed to shed more light on these relationships.

Some evidence has shown that early‐maturing women have higher fecundity (Udry & Cliquet 1982) which, if is the case in this sample, would again lead us to expect a relationship between age at menarche and total children. As with age at first birth, we find no direct or indirect link between these two variables. Previous studies have found associations between age at menarche and age at first birth even in high income, modern‐contracepting populations (Udry & Cliquet 1982). The Newcastle sample fits this description as these women would have been maturing around 1962 (age 15), by which time the contraceptive pill had become available in the United Kingdom (although for married women only at that time). Similar positive relationships between age at menarche and age at first pregnancy have been found in US women born between 1924 and 1956, constituting a more comparable sample (Udry, 1979). It is therefore interesting that the results of our models do not support such previous research, and implies that the direct effects of social class and housing on age at first birth are likely to be mediated by other unmeasured factors in this study. However, note that the housing grade scale, as measured by the Newcastle City Authorities, does not have too much variation in this sample (see Table 1), so it is interesting that there is evidence for a significant impact of housing on total number of children; more variation in housing conditions may have produced even stronger results. Our findings do not support other studies that have shown a positive relationship between height and age at first birth (Helle, 2008; Stulp et al., 2012; Sear et al., 2004). This is likely to reflect a trade‐off between growth and reproduction where taller women have invested longer in growing and end up entering into motherhood later than girls who stopped growth sooner. Therefore, it is surprising that while age at menarche was associated with height in this study, the path terminates here, and we also do not see a positive correlation between height and number of children.

None of our proposed mediating variables correlated with either reproductive outcome, although we can see from Figure 3 that age at first birth is the strongest predictor of number of children. The main difference between the two models is the relationship between SES and age at first birth but not total children, while poor housing remains significant for both. Perhaps the lack of evidence for an association between early life socioeconomic disadvantage and number of children reflects that, while these two reproductive variables can be considered part of a suite of life history events, this does not mean they are necessarily affected by the same early life conditions, particularly in a low fertility, modern‐contracepting population.

If childhood adversity affects lifetime health, which in turn affects longevity, we would expect early reproduction to be the adaptive response due to the individual's life expectancy being limited (Nettle et al., 2013b; Rickard et al. 2014). Perhaps, then it is surprising that childhood adversity was not associated with child health outcomes (birth weight and ailments in particular). Again this is likely to be a consequence of the post‐war food rationing system that may have evened out nutritional disparities between socioeconomic groups (Kuh et al. 2002). Furthermore, the British National Health Service (NHS) was established in 1948, providing free healthcare for everyone, reducing infant mortality during this period and making it likely that poor health was somewhat mitigated by access to good medical care in this population. Alternatively, our sample may have relatively limited power to detect effects. It is relatively small and also somewhat biased in that we only have information for people who were still alive at age 50, and for women who had children. Our sample was, however, sufficiently powerful to detect direct links between SES and age at first birth, between SES and child growth, and between age at first birth and total number of children. This suggests that, even if there are mediating links from SES to fertility through childhood physiological traits that we are unable to detect, they are somewhat weaker than other pathways. Furthermore, as can be seen in the bivariate correlation matrix (Table 3), the nonsignificant correlations are very small and so are unlikely to be solely an issue of sample size. Future research would help to identify the relevant pathways if they are able to include indicators of psychosocial stress, as well as physiological and health markers within the same population. We caution also that our findings here may not be generalizable because our data were limited by restricting our analysis to only those women who completed their fertility histories in the 1997 follow‐up survey. Future research using a larger sample with full information on completed fertility will be able to test if these findings are representative of the wider population.

## CONCLUSION

We found direct links between childhood social class, and childhood housing conditions, and age at first birth. We also show that even in a sample with access to effective contraception, age at first birth completely mediates the relationship between early life adversity and total numbers of children. As far as we are aware, this has not been shown before. It is surprising that age at menarche, and attained adult height, was not significantly associated with either age at first birth or total reproductive output as these factors are often taken to be part of a single life history trajectory. It is possible that psychosocial traits are more important mediators in the relationship between childhood adversity and reproduction than are physiological ones, at least in relatively well‐nourished population such as the one we study, and we encourage further research using data that are able to test this hypothesis. This study highlights the importance of uncovering the pathways between early life conditions and reproductive outcomes in later life because it is still unclear if all reproduction‐related outcomes, and their antecedents, are indeed part of a life‐history suite of events, as predicted by LHT.

## Supporting information

Supporting Information
